# Feministische Perspektiven in der deutschsprachigen Medizinethik: eine Bestandsaufnahme und drei Thesen

**DOI:** 10.1007/s00481-022-00724-8

**Published:** 2022-10-13

**Authors:** Mirjam Faissner, Kris Vera Hartmann, Isabella Marcinski-Michel, Regina Müller, Merle Weßel

**Affiliations:** 1 Klinik für Psychiatrie, Psychotherapie und Präventivmedizin, LWL-Universitätsklinikum, Ruhr-Universität Bochum, Alexandrinenstraße 1–3, 44791 Bochum, Deutschland; 2 Institut für Geschichte und Ethik der Medizin, Medizinische Fakultät, Ruprecht-Karls-Universität Heidelberg, Heidelberg, Deutschland; 3 Institut für Ethik und Geschichte der Medizin, Medizinische Fakultät, Georg-August-Universität Göttingen, Göttingen, Deutschland; 4 Institut für Philosophie, Universität Bremen, Bremen, Deutschland; 5 Ethik in der Medizin, Department Versorgungsforschung, Fakultät VI Medizin und Gesundheitswissenschaften, Carl von Ossietzky Universität Oldenburg, Oldenburg, Deutschland

**Keywords:** Gerechtigkeit, Intersektionalität, Literaturreview, Diskriminierung, Gender, Feminismus, Justice, Intersectionality, Literature review, Discrimination, Gender, Feminism

## Abstract

Im internationalen Diskurs sind feministische Perspektiven auf die Medizinethik bereits etabliert. Demgegenüber scheinen diese bislang nur vereinzelt in den deutschsprachigen medizinethischen Diskurs eingebracht zu werden. In diesem Artikel untersuchen wir, welche feministischen Perspektiven im deutschsprachigen medizinethischen Diskurs vertreten sind, und schlagen weitere Ansätze für eine feministische Medizinethik vor.

Zu diesem Zweck zeichnen wir mittels einer systematisierten Literaturrecherche feministische Perspektiven im deutschsprachigen medizinethischen Diskurs seit der Etablierung der Medizinethik als eigenständiger institutionalisierter Disziplin nach. Wir analysieren, welche Themen bereits innerhalb der Medizinethik aus einer feministischen Perspektive untersucht worden sind, und identifizieren Leerstellen. Basierend auf der Literaturrecherche, unseren eigenen Vorarbeiten sowie der Zusammenarbeit in der Arbeitsgruppe in der Akademie für Ethik in der Medizin „Feministische Perspektiven in der Bio- und Medizinethik“ stellen wir drei Thesen vor, die aus unserer Sicht einer Weiterentwicklung des deutschsprachigen medizinethischen Diskurses dienen können. Die erste These bezieht sich auf die Ziele feministischer Medizinethiken und besagt, dass diese (epistemische) Gerechtigkeit anstreben. Die zweite These stellt zentrale Eigenschaften von feministischen Medizinethiken als kritisch und kontext-sensibel heraus. In der dritten These diskutieren wir Intersektionalität und Postkolonialismus als theoretische Ansätze, die zu einer epistemisch gerechten, kritischen und kontext-sensiblen Medizinethik beitragen können. Wir argumentieren, dass feministische Perspektiven grundständig verankert werden sollten. Der Artikel schließt mit einem Ausblick auf die Arbeit der im letzten Jahr gegründeten Arbeitsgruppe in der Akademie für Ethik in der Medizin „Feministische Perspektiven in der Bio- und Medizinethik“.

## Abstract (en)

**Definition of the problem:**

Feminist approaches to medical ethics are well established in international discourses. By contrast, in the German-speaking medical ethical discourse, they still seem to be rather marginal. In this article, we analyze which feminist perspectives are prominent in German medical ethics and suggest new approaches.

**Arguments:**

We present our results from a systematized review of the literature, in which we identify existing feminist approaches within the German-speaking medical ethics discourse as well as research gaps. Based on the review, our preliminary research and discussions in the working group “Feminist perspectives in bio and medical ethics”, we defend three hypotheses aimed at advancing the German feminist medical ethical discourse. We posit that (1) feminist medical ethics aim at (epistemic) justice, (2) feminist medical ethics are critical and context-sensitive, and (3) intersectional and postcolonial approaches within feminist medical ethics may contribute to epistemically just, critical, and context-sensitive medical ethics.

**Conclusion:**

We argue that feminist perspectives should be implemented as a fundamental perspective in medical ethics because they can bring together key dimensions such as epistemically just, critical, context-sensitive, intersectional, and postcolonial thinking.

## Einleitung

Feminismus wird im gesellschaftlichen Diskurs oft als eine einheitliche Bewegung zur „Emanzipation der Frau“ verstanden. Dieses Verständnis greift allerdings zu kurz, da es bereits früh verschiedene Ausdifferenzierungen gab. In wissenschaftlichen Publikationen lassen sich beispielsweise *Differenzfeminismus, dekonstruktivistischer Feminismus, Queer-Feminismus, Ökofeminismus, materialistischer Feminismus, intersektionaler Feminismus, Schwarzer Feminismus *und* liberaler Feminismus *differenzieren. Angesichts dieser multiplen feministischen Strömungen, die eine Bezeichnung als „Feminismen“ angemessener erscheinen lässt (Pöge et al. 2014), ist die Angabe einer übergeordneten Definition ein anspruchsvolles Unterfangen. In einer ersten Annäherung verstehen wir Feminismus als eine Bewegung, die die Überwindung ungleicher Verhältnisse anstrebt, die aufgrund von vergeschlechtlichenden patriarchalen, cis-heteronormativen und sexistischen Strukturen entstehen (Grabow 2021). Hierbei berücksichtigen wir, dass diese Strukturen ihre Wirkmacht nicht isoliert entfalten, sondern konstitutiv mit weiteren Unterdrückungsstrukturen, wie rassistischen oder klassistischen Strukturen, verwoben sind (Haslanger 2020).

Als Querschnittsforschung diverser geistes- und sozialwissenschaftlicher Disziplinen hat feministische Forschung gezeigt, dass vergeschlechtlichte soziale Normen Menschen benachteiligen. Darüber hinaus hat feministische Forschung entscheidend dazu beigetragen, die Rechtfertigungssysteme ungerechter gesellschaftlicher Praktiken grundlegend zu hinterfragen, indem sie Hintergrundannahmen dekonstruiert hat. So zeigte post-strukturalistisch orientierte und queer-feministische Forschung, dass viele der gegenderten sozialen Normen auf einem wissenschaftlich nicht haltbaren Geschlechter-Binarismus sowie einer homogenisierten Vorstellung der Kategorie „Frau“ beruhen, die Menschen marginalisieren, die sich nicht innerhalb der binären Kategorien „Mann“ und „Frau“ definieren (Butler 2011). Intersektionale Forschung zeigte, dass soziale Gendernormen zudem mit anderen sozialen Kategorien, wie *Race* oder Klasse überschnitten (Intersektionen) sind, die ebenfalls mit Marginalisierungen einhergehen können (z. B. Crenshaw 1989). Feministische Forschung bietet somit Analyse-Instrumente, die einer Medizinethik, die soziale Normen und Praktiken kritisch hinterfragt, dienen können.

Die Medizinethik bezieht ihre Reflexionen vornehmlich auf die Medizin und Gesundheitssysteme, also auf Disziplinen bzw. Praxen, die immer noch stark von patriarchalen und cis-heteronormativen Strukturen geprägt sind. Frauen*^1^ sowie non-binäre, trans* und inter* Personen erfahren in der Medizin und in Gesundheitssystemen Benachteiligungen. Ein Beispiel ist das Yentl-Syndrom, das Phänomen, dass Frauen* Fehldiagnosen und -behandlungen erhalten, wenn ihre Symptome oder Erkrankungen nicht denen der Männer entsprechen (Merz 2011). Ein weiteres Beispiel ist die generell schlechtere gesundheitliche Situation und Versorgung von Schwarzen^2^ Frauen*, etwa mit Blick auf Herzerkrankungen (American Heart Association 2019), oder von non-binären, trans* und inter* Personen im Rahmen der Covid-19-Pandemie (Wang et al. 2020). Eine explizit feministische Perspektive scheint umso relevanter, da aktuelle technologische und digitale Entwicklungen in der Medizin, etwa auf selbstlernende Algorithmen gestützte Systeme, oft auf genderbasierten Datenlücken aufbauen. Diese bringen das Risiko mit sich, diese Lücken und somit Diskriminierungen zu perpetuieren (Cirillo et al. 2020). Es bleibt also weiterhin wichtig, auf diese ungerechten Strukturen aus der Perspektive von Frauen* hinzuweisen. Aus diesem Grund halten wir eine explizit feministische Perspektive auf medizinethische Themen für notwendig und angemessen.

Wie andere wissenschaftliche Disziplinen verfügt die Medizinethik über einen differenzierten Diskurs, der neben Fachtagungen und Konferenzen vorrangig in deutsch- und englischsprachigen Fachzeitschriften, wie *Ethik in der Medizin* oder *Bioethics*, geführt wird. Dieser Diskurs ist zentral für die Weiterentwicklung medizinethischen Wissens und medizinethischer Theorien, da hier wichtige innerdisziplinäre Fachdebatten geführt werden. Dabei geht es sowohl um aktuelle Fragestellungen rund um technologische oder politische Entwicklungen wie auch um theoretisch-begriffliche Ausdifferenzierungen und Klärungen, die für die Qualität medizinethischer Theoriebildung zentral sind. Welche Positionen innerhalb dieses medizinethischen Diskurses vertreten sind, hat damit einen großen Einfluss auf die inhaltliche, theoretische und methodologische Weiterentwicklung des Faches.

Aufgrund der disziplinären Sichtbarkeit von Positionen ist relevant, welche Debatten in welchen Kontexten geführt werden. Im deutschsprachigen Raum werden unterschiedliche feministische Debatten geführt, die auch für die Medizinethik von Relevanz sein können, z. B. im Bereich des Post- und Transhumanismus (Loh 2019), der feministischen Wissenschafts- und Technikforschung (Ernst 2013) oder der feministischen Pflegeethik (Kohlen und McCarthy 2020). Dabei ist auffallend, dass diese explizit feministischen Debatten vorranging in Fachzeitschriften für feministische Philosophie oder in Gender-wissenschaftlichen Zeitschriften geführt werden und in den medizinethischen Fachzeitschriften weniger präsent sind. Die Gründe für das scheinbare Ausweichen feministisch-medizinethischer Forschung auf Publikationsmedien außerhalb der Medizinethik mögen vielfältig sein und könnten sowohl in den Entscheidungen der Autor*innen als auch in denen der Editor*innen liegen. Gleichwohl scheint uns, dass durch eine mögliche Marginalisierung des feministischen Diskurses innerhalb der Medizinethik eine kritische Auseinandersetzung mit feministischen Perspektiven auf medizinethische Fragestellungen erschwert sein könnte. Daraus ergibt sich die Forschungsfrage, welche feministischen Perspektiven auf medizinethische Themen *innerhalb* des deutschsprachigen medizinethischen Diskurses vertreten sind. Diese diskutieren wir im ersten Teil des Artikels durch die Vorstellung einer systematisierten Literaturrecherche, in der wir uns explizit auf medizinethische Fachpublikationen beschränken, um eine mögliche Marginalisierung feministischer Diskurse in diesen Medien zu untersuchen. Im zweiten Teil des Artikels lösen wir uns von der empirischen Analyse des aktuellen Forschungsstandes und wenden uns Perspektiven zu, mit denen eine feministische Medizinethik weiterentwickelt werden kann. Basierend auf eigenen Vorarbeiten, der Literaturrecherche sowie der Arbeit innerhalb der Arbeitsgruppe in der Akademie für Ethik in der Medizin (AEM) „Feministische Perspektiven in der Bio- und Medizinethik“ (FME) stellen wir drei Thesen vor, die einer Weiterentwicklung des medizinethischen Diskurses im deutschsprachigen Raum dienen können. Dabei erörtern wir die Relevanz der feministischen Themen der 1) epistemischen Gerechtigkeit, 2) Kontext-Sensitivität und 3) Intersektionalität für den medizinethischen Diskurs. Wir schließen mit Implikationen für den allgemeinen medizinethischen Diskurs sowie einem Ausblick auf die Arbeit der AG FME.

## Bestandsaufnahme: Feministische Perspektiven in der deutschsprachigen Medizinethik

### Methodisches Vorgehen

Um feministische Ansätze und Perspektiven in der deutschsprachigen Medizinethik seit der Entwicklung der Medizinethik als institutionalisierter Disziplin nachzuzeichnen, haben wir in Kooperation mit der Dokumentationsstelle Ethik in der Medizin (IDEM) eine systematisierte Literaturrecherche in der Datenbank ETHMED durchgeführt. In ETHMED werden medizinethische Publikationen aus dem deutschsprachigen Raum in deutscher und englischer Sprache seit 1993 erfasst und verschlagwortet. Wir führten eine Suche von 1993 bis 2021 zu den Schlagwörtern „feminism“, „feminist“ oder „gender“ durch. Wir haben uns in der Recherche auf diese Schlagwörter konzentriert, um möglichst fokussierte Ergebnisse zu erhalten. Die Schlagwörter haben sich im Verlauf unserer Vorarbeiten herauskristallisiert: Sie wurden in einer ersten explorativen Suche getestet, im Autorinnenteam diskutiert und entsprechend begrenzt. Durch die Wahl der und Begrenzung auf die Suchwörter „feminism“, „feminist“ und „gender“ zielten wir auf Publikationen, die sich explizit als feministisch begreifen.^3^

Darüber hinaus durchsuchten wir die Datenbanken der deutschsprachigen Zeitschriften *Ethik in der Medizin, Medizinische Ethik, Bioethica* und *SGBE Bulletin SSEB* mit denselben Schlagwörtern. Um auch englischsprachige Beiträge von Autor*innen, die explizit auf den feministischen medizinethischen Diskurs im deutschsprachigen Raum Bezug nehmen, einzuschließen, führten wir eine ergänzende Recherche in den Datenbanken der Zeitschriften *Journal of Medical Ethics, International Journal of Feminist Approaches to Bioethics, American Journal of Bioethics* und *Bioethics *mit den Suchbegriffen (″feminist″ OR ″feminism″) AND ″German″ durch. Zusätzlich sichteten wir in der Universitätsbibliothek Göttingen per Handsuche Beiträge mit feministischen Bezügen in den Handbüchern zur Einführung in die Medizinethik, sowie in Handbüchern im Bereich Geschichte, Ethik und Theorie der Medizin.

Unser Screening basierte auf im Verlauf der Recherche definierte Ein- und Ausschlusskriterien. Wir schlossen alle medizinethischen wissenschaftlichen Artikel mit Peer-Review Verfahren, Artikel in Sammelbänden, Editorials und Kommentare ein, die von mindestens einem*r Autor*in aus dem deutschsprachigen Raum verfasst worden sind, eine feministische Perspektive einnehmen und in medizinethischen Zeitschriften oder Sammelbänden veröffentlicht worden sind. Dabei verstanden wir Positionen als feministisch, die im Text selbst entweder explizit als „feministisch“ oder „feminist“ bezeichnet werden, ein einschlägiges feministisches Framework nutzen, oder die kritisch Genderrollen und -normen oder patriarchale Machtstrukturen in die Analyse einbeziehen.^4^ Als medizinethisch verstanden wir Beiträge, die ethische Fragen in der Medizin und im Gesundheitswesen untersuchen.

Die Recherche in ETHMED ergab für den Zeitraum von 1993 bis 2021 insgesamt 218 Treffer. Zusätzlich identifizierten wir 151 Artikel über die Suchmaschinen der einzelnen Zeitschriften und durch die Handsuche. Nach einem Screening von zunächst den Titeln und anschließend den Abstracts durch die Autorinnen verblieben 73 Referenzen. Bei Unsicherheiten bzgl. eines Einschlusses wurden die Publikationen unter Vorbehalt aufgenommen. Die 73 Publikationen wurden dann nach dem Zufallsprinzip den Autorinnen zugewiesen, die die Volltexte der ausgewählten Literatur sichteten und Publikationen ausschlossen, die die Einschlusskriterien eindeutig nicht erfüllten. Dieses erste Volltextscreening enthielt noch unterschiedliche Bewertungen bzw. Unsicherheiten bzgl. einiger Publikationen. Ein wiederholtes Screening der verbleibenden Publikationen im Volltext durch eine andere Autorin, begleitet von Diskussionen unter den Autorinnen, führte zu einer Einigung auf die ausgewählten 27 Referenzen. Diese 27 Referenzen wurden in die finale Auswertung eingeschlossen und unterschiedlichen Themen zugeordnet (siehe Tab. 1).

**Table 1:** 

Thema	Anzahl	Aufsätze
Grundbegriffe feministischer Medizinethik	6	Nagl-Docekal und Pauer-Studer (1993); Vollmann (1995); Wirtz (1996); Pauer-Studer (1996); Haker (2003); Walser (2004)
Reproduktionsmedizin	6	Kuhlmann (1998); Wolbert (2002); Buse (2003); Kalendar (2010); Bernstein und Wiesemann (2014); Walser (2017)
Lebendorganspende	4	Schicktanz et al. (2010); Motakef (2013); Motakef und Wöhlke (2013); Beier und Wöhlke (2019)
Diskriminierung und Marginalisierung	4	Graumann (2003); Giese (2019); Hädicke und Wiesemann (2021); Faissner et al. (2022)
Forschungsethik	3	Wild (2007); Verdonk et al. (2020); Müller (2021)
Altersmedizin	2	Foth und Leibing (2021); Weßel (2021)
Geschlechtsidentität	1	Hester (2004)
Digitalisierung und Public Health	1	Sauerborn et al. (2021)

### Ergebnisse

Ein erstes Themenfeld widmet sich den theoretischen Grundlagen einer feministischen Medizinethik. Dabei zeigt sich, dass feministische Ansätze schon früh in der Medizinethik im deutschsprachigen Raum diskutiert worden sind, und zwar ausgehend von philosophischen Debatten zur feministischen Ethik. Bereits 1993 publizieren Nagl-Docekal und Pauer-Studer einen Sammelband, der unterschiedliche feministische Perspektiven auf die philosophische Ethik abbildet und im Rahmen dessen auch die Medizinethik adressiert. Diese Überlegungen werden durch eine Problematisierung der Grundbegriffe feministischer Ethik für medizinethische Fragen begleitet (Vollmann 1995). In Handbuchbeiträgen von Pauer-Studer (1996) und Haker (2003) werden Grundbegriffe feministischer Ethik, wie relationale Autonomie, Sorge und Gerechtigkeit, erläutert und in Bezug zu medizinethischen Debatten gesetzt. Weitere Autor*innen formulieren schließlich eine feministische Kritik des herkömmlichen bioethischen Diskurses (Walser 2004) und entwickeln ethische Grundanliegen einer feministischen Bioethik (Walser 2004) und Psychotherapie (Wirtz 1996).

Fragen reproduktiver Gerechtigkeit standen seit der Herausbildung einer feministischen Medizinethik auf der thematischen Agenda (Weßel et al. 2022). Auch im deutschsprachigen feministischen medizinethischen Diskurs werden reproduktionsmedizinische Fragen diskutiert, etwa in Bezug auf Schwangerschaftsabbruch (Kuhlmann 1998), In-Vitro-Fertilisation (Wolbert 2002) oder Hysterektomie (Buse 2003). Jüngere Publikationen widmen sich der Eizellspende und dem „social freezing“. So liegen Untersuchungen über „social freezing“ unter dem Aspekt von Altersdiskriminierung (Bernstein und Wiesemann 2014) sowie aus einer theologisch-feministischen Perspektive (Walser 2017) vor. Schließlich kritisiert Kalendar (2010), dass bislang Heteronormativität und Behinderung nicht systematisch und hinreichend in ethische Analysen reproduktiver Medizin einbezogen wurden, so dass queere Personen und Menschen mit Behinderung marginalisiert werden. Eine weitere queer-feministische Publikation beschäftigt sich mit ethischen Fragen um geschlechtsangleichende Operationen von inter* Kindern (Hester 2004).

Besondere Beachtung findet das Thema der Lebendorganspende. Dabei werden familiäre Reziprozitätsverhältnisse unter Berücksichtigung gegenderter sozialer Normen und Rollenverständnisse (Wöhlke 2014; Motakef 2013), auch im Vergleich zu sozialen Normen im Rahmen der Leihmutterschaft (Beier und Wöhlke 2019), in den Blick genommen. Weiterhin diskutieren Motakef und Wöhlke (2013) die Lebendorganspende aus einer marxistisch-feministischen Perspektive als eine Praxis der sozialen Reproduktion.

Untersuchungen zu forschungsethischen Fragen wenden sich dem Studieneinschluss von schwangeren Frauen (Wild 2007), der Forschung über sexuelle Orientierung und Identität vor dem Hintergrund heteropatriarchaler Strukturen (Müller 2021) sowie der Nutzung des Konzepts der Intersektionalität in palliativmedizinischer Forschung zu (Verdonk et al. 2020).

Neuere Arbeiten widmen sich innovativen feministischen Ansätzen. Im Bereich der Ethik der Geriatrie schlagen Foth und Leibing (2021) eine neue Konzeptualisierung von Demenz unter Rückgriff auf die Queer Theory vor. Weßel (2021) argumentiert für eine intersektionale Erweiterung geriatrischer Assessments. Zudem werden Aspekte struktureller und epistemischer Ungerechtigkeit von Gewalt gegen Frauen* unter einer Public Health Perspektive untersucht (Sauerborn et al. 2021). Fragen epistemischer Ungerechtigkeit finden auch im Kontext psychischer Erkrankung Beachtung (Faissner et al. 2022). Darüber hinaus werden auch der Begriff der Diskriminierung für die Medizinethik (Hädicke und Wiesemann 2021) sowie das Konzept der Unterdrückung für die Analyse von Arbeitsverhältnissen in der Pflege (Giese 2019) fruchtbar gemacht.

### Diskussion und Limitation

Insgesamt weist unsere Recherche darauf hin, dass im deutschsprachigen feministischen medizinethischen Diskurs bereits zahlreiche Themen und Ansätze, wie Intersektionalität oder Crip Theory^5^, vertreten sind (vgl. Kalendar 2010). Innerhalb des medizinethischen Diskurses fällt eine thematische Fokussierung und Verdichtung auf reproduktionsmedizinische Fragen sowie ethische Fragen rund um die Lebendorganspende auf. Vor allem in den letzten zehn Jahren zeigt sich jedoch eine Diversifizierung von Ansätzen und Themen, zum Beispiel in Bezug auf medizinethische Fragen im Bereich der Altersmedizin, zu Diskriminierung und Marginalisierung oder forschungsethischen Fragen. Gleichzeitig kann festgehalten werden, dass noch zahlreiche thematische Leerstellen bestehen. So erhalten feministische Perspektiven auf medizinethische Fragen hinsichtlich Digitalisierung und Technik, Public Health oder Ethik in der Psychiatrie bislang nur sehr begrenzten Einzug in den deutschsprachigen medizinethischen Diskurs.

Unsere Suchstrategie weist einige Limitationen auf. Zunächst war unser Forschungsinteresse auf den Diskurs in medizinethischen Fachzeitschriften gerichtet. Darum erheben wir keinen Anspruch auf eine vollständige Erfassung des gesamten feministischen medizinethischen Diskurses. Darüber hinaus haben wir unsere Suchstrategie durch die Wahl der Suchwörter auf Artikel fokussiert, die sich explizit als feministisch begreifen. Wir halten es für möglich, dass Autor*innen feministische Arbeiten nicht explizit als „feministisch“ bezeichnen (vgl. Kalendar 2010). Solche implizit feministischen Beiträge zu medizinethischen Debatten haben wir in unsere Recherche nicht einbezogen. Dasselbe gilt für feministische Beiträge aus verwandten Bereichen wie der Pflegeethik oder der Seelsorge.^6^ Unsere Literaturrecherche gibt unter Beachtung dieser Limitationen einen Überblick darüber, welche explizit feministischen Positionen innerhalb des deutschsprachigen *Mainstreams* des medizinethischen Diskurses vertreten sind.

Dieser erste Teil des Artikels diente einer Reflexion des aktuellen deutschsprachigen medizinethischen Diskurses. Im Folgenden wenden wir uns möglichen weiteren Anknüpfungsstellen zwischen feministischen Diskursen und medizinethischen Fragestellungen zu. Dafür stellen wir drei Thesen über feministische Perspektiven in der Medizinethik auf, die unserer Ansicht nach einen Beitrag zur Weiterentwicklung des medizinethischen Diskurses im deutschsprachigen Raum leisten können. Diese haben wir basierend auf eigenen Vorarbeiten, der Literaturrecherche sowie in Zusammenarbeit mit der AG FME entwickelt und aufgrund ihrer besonderen Relevanz für die Medizinethik zur Diskussion ausgewählt. Die weitere Diskussion baut dabei insbesondere auf Ergebnissen und Forschung aus dem anglo-amerikanischen Raum auf, in dem feministische Perspektiven schon lange explizit etabliert und entsprechende Ansätze vorhanden sind.

## Drei Thesen zu feministischen Perspektiven in der Medizinethik

### These 1: Eine feministische Medizinethik strebt (epistemische) Gerechtigkeit an

Patriarchale und heteronormative Strukturen prägen nach wie vor alle Bereiche unserer Gesellschaft, inkl. des Gesundheitssystems, der medizinischen Praxis und Forschung sowie der Medizinethik. Eine feministische Medizinethik verschreibt sich dem Ziel, soziale Ungerechtigkeit aufzuzeigen und Gerechtigkeit zu fördern.

Viele Ansätze in der herkömmlichen Medizinethik erkennen Gerechtigkeit als einen zentralen Wert an. So fungiert Gerechtigkeit etwa als eines der vier grundlegenden bioethischen Prinzipien in dem verbreiteten Ansatz von Beauchamp und Childress (2001). In medizinethischen Diskursen wird Gerechtigkeit häufig im Sinne von Verteilungsgerechtigkeit verstanden und diskutiert, also hinsichtlich der Frage, wie Ressourcen im Gesundheitssystem gerecht verteilt werden können (Varkey 2021). Auch feministische Medizinethiken widmen sich Fragen von Verteilungsgerechtigkeit. Insbesondere bezogen auf Public Health kann eine feministische Perspektive die Zusammenhänge zwischen Geschlecht, Benachteiligung und Gesundheit sowie verschiedener Machtverhältnisse in Fragen von Verteilungsgerechtigkeit aufdecken (Rogers 2006). Unter Berücksichtigung gesellschaftlicher Verhältnisse, etwa dem Einfluss neoliberaler Ökonomisierungs- und Privatisierungsprozesse (Mohan 2018) auf Gesundheitssysteme, kann eine feministische Perspektive die Verteilung medizinischer Ressourcen reflektieren.

Eine weitere feministische Debatte hinsichtlich Gerechtigkeit betrifft die Benachteiligung von Personen bei der Produktion und Distribution von Wissen aufgrund struktureller Macht- und Herrschaftssysteme (Fricker 2007; Dotson 2012; Mills 2017). Im Kontext der Medizin zeigt sich dies zum Beispiel durch fehlendes Wissen über spezifisch weibliche Gesundheit aufgrund eines historisch dominanten Fokus auf den männlichen Körper, der durch patriarchale Strukturen in der Medizin geprägt ist. Eine Beschäftigung mit Fragen epistemischer Gerechtigkeit ist zentral für die Medizinethik als zum einen einer Institution der Wissensproduktion und -distribution selbst und zum anderen als Institution, die die Rolle eines Korrektivs für Forschung, also Wissensproduktion, in den Lebenswissenschaften übernimmt. International haben Fragen epistemischer Ungerechtigkeit bereits Einzug in medizinethische Debatten erhalten (Drożdżowicz 2021). Im deutschsprachigen Diskurs steht die Beschäftigung mit dem Thema noch in den Anfängen (Schicktanz 2012; Faissner et al. 2022; Sauerborn et al. 2021).

Ein wichtiger Aspekt epistemischer Gerechtigkeit liegt in der Wahl hermeneutischer Ressourcen, wie Theorien, Konzepte oder Wissenskörper, die dem Verständnis und Kommunizieren sozialer Erfahrungen dienen. Dem Diskurs um epistemische Gerechtigkeit liegt die Idee zugrunde, dass innerhalb einer Gesellschaft unterschiedliche Sets hermeneutischer Ressourcen verfügbar sind, die von unterschiedlich epistemisch positionierten Gruppen entwickelt werden (Dotson 2012). Ein Beispiel einer solchen hermeneutischen Ressource stellt das Konzept der rassistischen Mikroaggression dar. Der Begriff beschreibt kurze und alltägliche verbale und verhaltensbasierte Herabwürdigungen, die in (expliziten oder impliziten) negativen Vorurteilen gegenüber Mitgliedern marginalisierter Gruppen begründet liegen (Pierce 1970). Mikroaggressionen können auch in der klinischen Praxis auftreten (Freeman und Stewart 2018). In Bezug auf die klinische Ethik erscheint es wichtig, dass Ethikberater*innen Mikroaggressionen, etwa aufgrund von rassistischen oder sexistischen Stereotypen, in Beratungen erkennen und hierfür sensibilisieren können. Verkennt ein*e klinische*r Ethikberater*in z. B. eine rassistische Mikroaggression als eine Spannung aufgrund „kultureller Differenz“, erfährt die Person, die das Ziel der Mikroaggression ist, einen epistemischen Nachteil, da eine wichtige soziale Erfahrung, die der rassistischen Diskriminierung im medizinischen Setting, verzerrt wird.

Aus diesem Grund halten wir eine besondere Sensibilität bei der Wahl hermeneutischer Ressourcen für eine feministische Medizinethik als zentral. Dies betrifft auch die Wahl des reproduzierten Kanons durch die eigene Zitierpraxis in wissenschaftlichen Texten. Eine feministische Medizinethik sollte aktiv prüfen, ob sie durch die Wahl ihrer hermeneutischen Ressourcen Ausschlüsse produziert, um nicht zur Marginalisierung strukturell benachteiligter Gruppen beizutragen. In ihrer Analyse epistemischer Ungerechtigkeit weist Dotson (2012) darauf hin, dass epistemische Ungerechtigkeit häufig und meist unabsichtlich geschehe. Sie zu vermeiden stellt darum eine große Herausforderung dar, die eine aktive Haltung, Sensibilität und Bemühung erforderlich macht. Nach der Darstellung eines wichtigen Ziels feministischer Medizinethik wenden wir uns nun ihren zentralen Eigenschaften zu.

### These 2: Eine feministische Medizinethik ist kritisch und kontext-sensibel

Die feministische Bioethikerin Sherwin (1989) hat bereits 1989 argumentiert, dass feministische Theorie und Medizinethik in einer „natürlichen“ Verbindung zueinander stehen, da beide die Abstraktheit und Generalität von moraltheoretischen Ansätzen kritisieren. Diese Idee einer Verbindung ist bis heute aktuell, aber insbesondere im deutschsprachigen Forschungskontext gibt es nur wenige theoretische Grundlagenwerke zur Verzahnung von Feminismus und Medizinethik (z. B. Walser 2004; Pauer-Studer 1996; für eine Problematisierung siehe Vollmann 1995).

Die Medizinethik als angewandte Ethik fokussiert ethische Fragen in klinischen Kontexten und weist hierdurch eine höhere Kontext-Sensibilität als andere Ethiken auf, indem sie z. B. den Perspektiven des Individuums, insbesondere des*der Patient*in, einen gesteigerten Wert beilegt. Dabei bleibt der*die Patient*in im Rahmen medizinethischer Theorien, wie z. B. in der Prinzipienethik von Beauchamp und Childress (2001), allerdings meist ein stark individualisiertes Konstrukt. Dies zeigt sich insbesondere auch im deutschsprachigen Kontext der Medizinethik, welche oftmals zum Beispiel in Diskursen um Gerechtigkeit einen starken Fokus auf das Individuum legt und dabei strukturellen Ungerechtigkeiten, die bestimmte Personengruppen betreffen, nicht ausreichend Rechnung trägt (Prietl 2021). Auch wenn Beauchamp und Childress auf die fehlenden relationalen Aspekte in ihrem Ansatz hinweisen, ist der individualisierte Autonomiebegriff insbesondere für feministische Care-Ethiker*innen ein Ansatz für Kritik. So weist z. B. Robinson (2011) darauf hin, dass relationale Aspekte eine wichtige Rolle für ein Verständnis von Positionierungen des Individuums im Kontext von Machtstrukturen spielen und darüber hinaus auch als macht-kritisches Instrument dienen, um Ungerechtigkeiten sichtbar zu machen.

Insbesondere die empirisch-informierte Medizinethik, die sich in den vergangenen Jahren vor allem auch in der deutschsprachigen Medizinethik etabliert hat (z. B. Salloch et al. 2011; Schweda 2019), hat es sich zur Aufgabe gemacht, die Perspektiven von Individuen stärker kontext-sensibel in die medizinethische Forschung einzubeziehen (z. B. Seidlein et al. 2019). Die empirisch-informierte Medizinethik ist aus einer internen, wie auch externen Kritik an der mangelnden Praktikabilität, Durchsetzbarkeit und Kontextsensibilität medizinethischer Ansätze für die medizinische Praxis heraus entstanden (Musschenga 2009). Hier zeigen sich Überschneidungsmerkmale zur feministischen Ethik und ihren Forderungen nach höherer Kontextsensibilität sowie einer genaueren Betrachtung der Wechselwirkungen von sozialen und ethischen Normen.

Studien, die Methoden der empirisch-informierten Ethik nutzen, belegen, dass eine höhere Kontextsensibilität zu differenzierten Erkenntnissen führen kann. So zeigen z. B. Forschungen zur Organspende, dass Frauen* eine höhere Bereitschaft zur Organspende aufweisen, die sich aus einem moralischen Verpflichtungsgefühl oder der Norm, dass Frauen* vermehrt relational entscheiden, ableiten lassen könnte (Schicktanz et al. 2010; Beier und Wöhlke 2019; Motakef 2013; Motakef und Wöhlke 2013). Durch den empirisch-informierten Ansatz konnte das Geschlecht der organspendenden Person als eine wichtige Grundlage für die moralische Begründung für oder gegen eine Bereitschaft zur Organspende herausgearbeitet werden. Eine feministische Kritik dieser moralischen Begründung kann z. B. die patriarchalen Normen, die ihr zugrunde liegen können, weiter herausdifferenzieren und so zu einem umfassenderen Verständnis der Geschlechterdifferenz in der Organspende beitragen.

Nicht nur in ethischen Fragen zur Organspende, auch im Bereich der Digitalisierung zeigen kritische und kontext-sensible Ansätze hohes Erkenntnispotential. Vor allem neue technische Möglichkeiten in der Medizin und Pflege sollten aus einer feministischen Perspektive kritisch und kontext-sensibel begleitet, und ihre Chancen und Risiken insbesondere hinsichtlich ihrer machtvollen sozialen, psychologischen und leiblichen Auswirkungen untersucht und beurteilt werden (Sauerborn et al. 2021). Technische Artefakte sind nicht neutral, auch wenn insbesondere Technikentwickelnde oftmals das hohe Neutralitätspotenzial von Technologien hervorheben (Weßel et al. 2021). Die zuweilen diskriminierenden Auswirkungen entstehen in der Praxis und stehen in Wechselwirkung mit den Entwickler*innen einerseits und den Nutzer*innen andererseits. Entsprechend sollten die technischen Entwicklungen in ihren vielfältigen Auswirkungen auf, aber auch Vorannahmen über die Nutzer*innen, kritisch reflektiert werden (Kalendar 2010; Kuhlmann 1998; Weßel et al. 2021).

Ein kritischer und kontext-sensibler, feministischer Ansatz in der Medizinethik hat das Potenzial, bislang unbeachtete Aspekte aufzudecken und vorherrschende normative Annahmen kritisch zu hinterfragen. Darin liegt ein hohes Erkenntnispotential für ein besseres Verständnis moralischer Argumentationen sowie einen kritischen Umgang mit als selbstverständlich wahrgenommenen normativen Ansätzen.

In der letzten These argumentieren wir, dass neben den Methoden einer empirisch-informierten Medizinethik Intersektionalität und Postkolonialismus die Möglichkeit bieten, das Desiderat einer kritischen und kontext-sensiblen Medizinethik zu erreichen.

### These 3: Eine feministische Medizinethik arbeitet intersektional und postkolonial

Intersektionalität wurde in den 1970er-Jahren durch Schwarze feministische Aktivist*innen wie dem Combahee River Collective (1977) entwickelt und von der Rechtswissenschaftlerin Crenshaw (1989) in den akademischen Diskurs eingeführt. In den folgenden Jahrzehnten wurde Intersektionalität von unterschiedlichen Disziplinen und Autor*innen aufgegriffen, adaptiert, reflektiert und weiterentwickelt, so dass mittlerweile viele unterschiedliche intersektionale Ansätze nebeneinander bestehen (Hill Collins 2015; Rice et al. 2019).^7^ Intersektionalität stellt eine Kritik am bis dahin überwiegend aus einer *weißen *Perspektive gedachten Feminismus dar (Crenshaw 1989) und dient der Analyse von Ungleichheit produzierenden miteinander verschränkten sozialen Strukturkategorien. So kann die Diskriminierung einer Schwarzen Frau* nicht isoliert auf Sexismus oder Rassismus oder ihrer Kombination zurückgeführt werden. Vielmehr müssen Rassismus und Sexismus und damit verbundene Diskriminierungserfahrungen als ko-konstitutiv und miteinander verwoben verstanden werden. Diese Einsicht hat sich gerade in Zeiten der Covid-Pandemie mit Blick auf das besondere Risiko von Schwarzen sozio-ökonomisch benachteiligten Personen in den USA an Covid-19 zu versterben, bestätigt. Insbesondere in der englischsprachigen Medizinethik wurde diskutiert, wie diese besondere Vulnerabilität bei der ethischen Beurteilung von Risiko-Scores zur Verteilung von Beatmungsgeräten während der Covid-Pandemie Beachtung finden soll (Bradock 2021). Wie diese Debatte zeigt, stößt das Konzept der Intersektionalität in internationalen medizinethischen Diskussionen mittlerweile auf Interesse (z. B. Grzanka et al. 2016; Wilson et al. 2019; de Proost 2021). Insgesamt findet es jedoch noch kaum Berücksichtigung; vor allem in der deutschsprachigen Medizinethik wird es selten genutzt (Kalendar 2010; Verdonk et al. 2020; Weßel 2021).^8^ Dies birgt das Risiko unvollständiger Analysen sozialer Ungerechtigkeiten, die folglich nicht korrekt in ethische Erwägungen mit einbezogen werden können.

Ein Beispiel für eine intersektionale und postkoloniale Betrachtung ist die Reflektion konkreter sozialer Bedingungen, wie der Arbeitsverhältnisse in zunehmend neoliberal geprägten Gesundheitssystemen. Die Arbeit in den meisten Bereichen der Gesundheitsversorgung ist in hohem Maße vergeschlechtlicht (Auth 2021), beeinflusst von sozio-ökonomischen Voraussetzungen, wie auch von der Kategorie *Race*, und damit von sozialen Ungleichheiten geprägt (Robinson und Mahon 2011). Ein weiteres Beispiel sind Patient*innen an der Intersektion von Geschlecht und Behinderung, die häufig von intersektionalen Diskriminierungen betroffen sind, die ihre Gesundheitsversorgung maßgeblich beeinflussen können (Waldschmidt 2014). Eine feministische Medizinethik kann daher mit intersektionalen und postkolonialen Perspektiven die Auswirkungen verschränkter sozialer Ungleichheiten innerhalb des Gesundheitssystems untersuchen und berücksichtigen.

Ansätze und Forschung der Critical Race Theory haben im englischsprachigen und auch beginnend im deutschsprachigen Raum zu einer Hinterfragung des eurozentristischen und durch Rassismus und Sexismus geprägten philosophischen Kanons geführt (Leopold und Mateo 2019).^9^ Ähnliche Bestrebungen in der deutschsprachigen Medizinethik finden bis jetzt noch wenig Aufmerksamkeit. Gerade eine feministische Medizinethik sollte stärker bedenken, wer für wen spricht und welche ethischen Konflikte adressiert bzw. nicht adressiert werden (Mithani et al. 2021). Denn auch die feministische Medizinethik reflektiert ihre eigenen Fragestellungen, Konzepte und Argumentationen noch zu wenig hinsichtlich intersektionaler und postkolonialer Rahmenbedingungen. Intersektionale und postkoloniale Ansätze können einen kritischen Beitrag zu der eigenen Reflexion innerhalb der Medizinethik in Bezug auf Wissensproduktion liefern.

## Fazit: Feministische Perspektiven als grundlegende Bausteine einer Medizinethik

Da feministische Blickwinkel zentrale Dimensionen wie epistemisch gerechtes, kritisches, kontext-sensibles, intersektionales und postkoloniales Denken zusammenbringen, können wir abschließend zusammenfassen, dass feministische Perspektiven in der Medizinethik grundständig sein sollten. Wenn das Nichtbeachten bestimmter Dimensionen zu unvollständigen oder verzerrten Analysen führen kann, wie wir z. B. in der dritten These aufgezeigt haben, folgt, dass feministische Perspektiven von grundlegender Bedeutung sind. Aufgrund dieses grundlegenden Charakters sind feministische Perspektiven nicht nur eine beliebige Erweiterung, sondern bilden einen zentralen Baustein im Fundament medizinethischer Überlegungen.

Eine feministische Medizinethik erlaubt, eine kritische Perspektive auf gesellschaftliche Zusammenhänge und strukturelle Faktoren einzunehmen. Der Blick wird nicht (nur) auf die individuelle Ebene oder auf spezifische Themen gerichtet, sondern auf zugrunde liegende Strukturen – somit ist eine feministische Perspektive für alle medizinethischen Themen relevant. Es ist daher nicht ausreichend, wenn sich allein eine kleine Gruppe damit beschäftigt oder feministische Perspektiven nur am Rande thematisiert werden, wie es derzeit in den deutschsprachigen Medizinethik-Debatten der Fall ist.

Eine feministische Medizinethik betrachtet bestehende Strukturen kritisch und hinterfragt somit die vorherrschenden medizinethischen Theorien und Modelle. Indem sie das theoretische Gerüst für medizinethische Analysen (z. B. prinzipienorientierte Medizinethik, Utilitarismus) sowie deren Kontext (z. B. die privilegierte Situation einer *weißen* akademischen Medizinethik; gender-spezifische Ungleichheiten in der Forschung) hinterfragt und die Aufmerksamkeit auf neue Themen lenkt (z. B. Bedürfnisse marginalisierter Gruppen), nutzt und entwickelt sie zeitgemäße theoretische und analytische Frameworks (z. B. Relationale Autonomie; Intersektionalität).

Gleichzeitig bedarf es einer kritischen Auseinandersetzung mit feministischer Medizinethik, verbirgt sich doch hinter diesem Label ein sehr heterogenes Feld, welches in den deutschsprachigen Medizinethik-Debatten noch nicht sichtbar genug ist. Weitere Forschung, interdisziplinärer Austausch sowie eine Auseinandersetzung mit feministischen Positionen innerhalb des medizinethischen Diskurses und permanente Kritik sind auch künftig wichtig, um den Diskurs um eine feministische Medizinethik weiter voranzubringen und für die Medizinethik fruchtbar zu machen.

### Die Arbeit der Arbeitsgruppe „Feministische Perspektiven in der Medizin- und Bioethik“ in der AEM

Für diesen Austausch möchte die Arbeitsgruppe „Feministische Perspektiven in der Medizin- und Bioethik“, die Anfang 2021 unter dem Dach der AEM von uns Autorinnen gegründet wurde, eine Plattform anbieten. Die Arbeitsgruppe soll die Möglichkeit bieten, gemeinsam feministische Perspektiven in der Medizinethik im deutschsprachigen Raum zu thematisieren, zu kritisieren, weiterzuentwickeln und systematisch zu verankern. Ging es beim Auftakttreffen der Arbeitsgruppe vor allem um die Fragen, was unter einer feministischen Medizinethik verstanden werden kann, welche Ziele und was mögliche Zusammenarbeiten einer solchen Arbeitsgruppe sein könnten (Hädicke 2021), wurden die Themen und Ziele der Gruppe bereits auf weiteren Treffen konkretisiert. Die Arbeitsgruppe plant, sich regelmäßig dreimal im Jahr zu treffen. Alle Interessierten sind herzlich willkommen!

## Footnotes

1 Der Begriff „Frauen*“ bezeichnet alle Personen, die sich selbst als Frauen identifizieren.

2 Wir verwenden den Begriff „Schwarz“ im Sinne der politischen Selbstbezeichnung, die eine sozio-politische Positionierung in einer *weiß *dominierten Gesellschaft markiert.

3 Die Suchbegriffe „Geschlecht“ und „sex“ erwiesen sich nicht als hinreichende Kriterien für einen Einschluss von Artikeln in unser Sample, da die Begriffe zumeist nur als deskriptive, soziodemographische Kategorien verwendet werden.

4 Artikel, die nach der Sichtung zwar feministischen Positionen zugeordnet werden können, dies aber nicht explizit benennen, wie z. B. Körtner (2008), Kohlen (2019) und Schneider (2021), wurden nicht in die Analyse mit einbezogen.

5 Crip Theory bezeichnet kritische Ansätze, die innerhalb der Disability Studies entwickelt worden sind (vgl. Kalendar 2010).

6 Für einen aktuellen Beitrag im Bereich der feministischen Pflegeethik siehe z. B. Kohlen (2020).

7 Für eine kritische Auseinandersetzung mit dem deutschsprachigen Diskurs in Bezug auf Intersektionalität, siehe Petzen (2012).

8 Wir verweisen auch auf den Artikel von Weßel und Schweda „Recognizing the diverse faces of later life. Old age as a category of intersectional analysis in medical ethics“, der voraussichtlich 2023 im Journal of Medicine and Philosophy erscheinen wird.

9 In diesem Kontext zu nennen sind auch neuere Untersuchungen über die Verstrickungen der Medizin(ethik) in der Kolonialgeschichte (z. B. Russell 2016).
